# Supplementary material for: A limbic circuit selectively links active escape to food suppression
Source: eLife. 2020 Sep 7;9:e58894. doi: 10.7554/eLife.58894 (PMC7476759; doi:10.7554/eLife.58894)
Supplement: Supplementary file 1. — Expression is noted as − (no expression), + (low expression), ++ (moderate expression) and +++ (high expression). [file elife-58894-supp1.docx]

**Table 1. c-Fos expression in the mouse brain after restraint stress.**

| **Brain Region** | **c-Fos expression** |
| --- | --- |
| Central Amygdala (CeA) | + |
| Basolateral Amygdala (BLA) | ++ |
| Insular Cortex (IC) | ++ |
| Claustrum (CLA) | +++ |
| Lateral Hypothalamic Area (LHA) | +++ |
| Lateral Habenula (Lhb) | ++ |
| Paraventricular nucleus of the Thalamus (PVT) | ++ |
| Lateral Septum (LS) | +++ |
| Medial Preoptic Nucleus (MPO) | +++ |
| Periqueductal Gray (PAG) | ++ |
| Bed Nucleus of the stria terminalis (BNST) | +++ |

**Score:** - (no expression), + (low expression), ++ (moderate expression), +++ (high expression)
